# What makes a good general practice consultation? An exploratory pilot study with people from a low socioeconomic background

**DOI:** 10.3399/BJGPO.2023.0160

**Published:** 2024-06-12

**Authors:** Naomi MacPherson, Binh Ta, Lauren Ball, Nilakshi Gunatillaka, Elizabeth Ann Sturgiss

**Affiliations:** 1 School of Primary and Allied Health Care, Monash University, Melbourne, Australia; 2 Centre for Community Health and Wellbeing, School of Public Health and School of Human Movement and Nutrition Sciences, The University of Queensland, Brisbane, Australia

**Keywords:** health inequities, general pracitce, qualitative research

## Abstract

**Background:**

While patients from low socioeconomic (SES) backgrounds are at increased risk of developing chronic health conditions, typically managed within general practice, they report fewer positive consultation experiences with GPs than patients from higher SES groups. To our knowledge, existing research does not provide an in-depth understanding of the GP conducts that contribute to positive consultations.

**Aim:**

To identify the factors that patients from low SES backgrounds perceive as essential for creating good consultation experiences.

**Design & setting:**

This exploratory pilot study was performed in GP clinics in Melbourne, Australia.

**Method:**

We used an appreciative inquiry approach, focused on positive consultation experiences, previously shown to be helpful for researching sensitive topics. Nine patients from low SES backgrounds, who reported positive consultation experiences, undertook a semi-structured qualitative interview while watching the video recording of their GP consultation. Four different GPs were captured in the recordings. Inductive thematic coding was performed by two researchers.

**Results:**

The following four categories were developed: 1) the doctor’s demeanour and how the patient was made to feel during the consultation drove their engagement; 2) an established and collaborative therapeutic relationship was of high importance to patients; 3) a doctor’s therapeutic skillset was integral to patient confidence and comfort; and 4) patients appreciated verbal and non-verbal communication techniques. In each interview, the discussion about the video-recorded consultation often triggered reflections about previous consultations with the respective GP.

**Conclusion:**

For patients from low SES groups, positive consultation experiences were underpinned by perceived continuity of care with a specific GP who consistently showed good communication skills and key interpersonal characteristics. This research is a small step towards increasing our understanding of the experience of individuals from low SES backgrounds in primary care and the existing health inequities within this area.

## How this fits in

Patients from low socioeconomic (SES) backgrounds have been shown to have poorer experiences within general practice consultations compared with patients from higher SES groups. High-quality therapeutic relationships have been shown to increase the likelihood of patients from low SES groups seeking and using primary health care effectively. Therefore, this pilot study utilised an appreciative inquiry approach to provide some small increases in our understanding of what makes a positive consultation experience for patients from low SES groups.

## Introduction

Health inequity refers to unfair and avoidable differences in health outcomes, secondary to social and structural determinants.^1,2^ Such social determinants of health include ethnic group, geographic location, education level, and SES.^1,2^ A 2016 Australian Institute of Health and Welfare report identified a 2.6 increased risk of diabetes, and a 2.2 increased risk of both stroke and coronary heart disease in in the lowest SES group compared with the highest.^3^ Therefore, Australia is no exception to the known social gradient of health.

General practice is often tasked with providing comprehensive care for patients from low SES backgrounds, especially regarding chronic disease management.^4^ The World Medical Association sees patient-centred, high-quality consultations as fundamental to *'meeting the needs of disabled and vulnerable groups'*.^5^ Yet we know from patient surveys by the Australian Bureau of Statistics,^6^ and international literature,^7–9^ that patients from low SES groups report fewer positive experiences within GP consultations compared with patients from higher SES backgrounds.

The association between SES and patient satisfaction may relate to clinician factors. This includes doctors’ negative perceptions,^10,11^ doctors displaying low empathy,^12^ or providing few explanations regarding diagnoses and results.^13,14^ Consultation length is positively correlated to patient satisfaction, yet in geographic areas of disadvantage, GP consultations are shorter than in more affluent areas.^15,16^ It is possible that in longer consultations, doctors spend more time exchanging information, educating, and advising about preventive health behaviour.^17^ Yet the patient’s perspective needs to be considered when trying to accurately ascertain the drivers that influence the quality of healthcare delivery.^18^

Existing research does not provide an in-depth understanding of the patient experience of individuals from low SES groups,^19^ nor does it pinpoint what GP conducts can result in a positive consultation. By using video-stimulated-recall interviews, this study can explore the experience of this patient group and provide greater insight into GP consultation practices with them. This could thereby provide some small increases in our understanding of ways to bridge the current gap in the delivery of health care to patients from low SES groups in general practice. This is important because it has been shown that higher-quality therapeutic relationships increase the likelihood of patients from low SES groups seeking and using primary health care effectively.^20^ Therefore, the aim of this research study was to understand the GP and consultation factors that patients from low SES groups perceive as essential for creating a good consultation experience.

## Method

**Figure 1.**
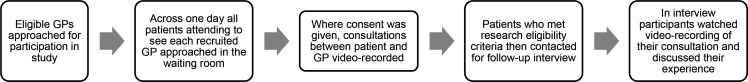
Schematic presentation of the data collection process

We undertook descriptive qualitative research, focused on in-depth interviews with patients who self-identified as low SES with chronic health conditions, while they watched and reflected on a video-recording of their GP consultation. The interviews conducted with eligible patients was the primary data collected. See Figure 1 for an overview of the participant recruitment and data collection processes.

We utilised an appreciative inquiry approach,^21^ which involves identifying components of successful situations and positive experiences to constructively frame what can be improved.^21,22^ This approach offers a deep understanding of the delivery of highly effective health care and results in contextualised information on best practices, which can then be implemented in other settings. Participants are more willing to talk in detail about their experience when discussing positive situations.^21^ Focusing on how and why things work will extend our understanding of how consultations can contribute to the health and wellbeing of those from low SES backgrounds.

The Hawthorne effect^23^ is often raised in research involving recorded consultations, with concerns regarding altered behaviour by participants owing to an awareness that they are being recorded. However, one study of US primary care found little Hawthorne effect from an in-person observer,^24^ and another found that audio-recording consultations did not introduce a significant Hawthorne effect on doctor–patient communication.^25^ Hence, this confirms the valuable contribution of real-life recordings for empirical research.

### Participant recruitment

As the interest for this project lay in exemplary consultation practices, we approached teaching and research GP clinics, alongside those that have been provided awards for excellence. From such clinics, GPs were recruited using the modified Dillman method.^26^ Invitations for participation were also disseminated via our Twitter (now X) account, and word-of-mouth recommendations (snowballing) were encouraged. Interested GPs completed a demographic survey and provided consent to having their consultations video-recorded.

From August 2021–February 2022, a researcher then visited practices where consented GPs worked, to recruit patients. At each clinic, patients attending to see the consented GP were informed by reception about the research occurring, before being approached by a researcher in the waiting room. The researcher explained the key purpose of our research and how we wanted to video-record GP patient consultations. For the approximately 50% of patients who indicated interest in being involved, the researcher then obtained their demographic details, written consent to be video-recorded, and permission to be contacted for a post-consultation interview. Following the video-recording of their consultations, patients filled out a paper-based survey about their experience with the GP (see Supplementary Information S1). These questions were based on the Australian Bureau of Statistics patient experience survey.^27^

### Data collection

The eligibility criteria to be interviewed were patients who had self-reported as low income with a chronic health condition, as well as reporting that they had felt listened to, respected, and had been given adequate time (yes, no, or unsure). No more than three patients per GP were interviewed.

In the interview, eligible patients viewed their recorded consultation and provided commentary on their experience. The interviews were conducted with an appreciative inquiry approach^21^ by an expert researcher specialising in applied linguistics and communication analysis. They emphasised to interviewees that they were interested in the specifics of how the GP acted and communicated to create a positive experience. Interviewees were encouraged to pause the video-recording when they wanted and to provide comments and insight. When the patients didn’t stop the video for approximately 3 minutes, the researcher paused the video and participants were asked, '*What has happened so far that you think was really good?'* See Supplementary Information S2 for further questions asked by the researcher. The interviews were audio-recorded.

### Data storage

All GP and patient survey-based data were identified. These identified data were given unique identifiers and coded. As per Monash University’s data management policies, the coded data were kept on a locked, password-protected document, only accessible by EAS.

### Data analysis

Interview audio-recordings were automatically transcribed (Otter.ai) verbatim. A research assistant double-checked the automated transcripts and de-identified them.

Two researchers performed inductive thematic coding and analysed the data using NVivo (version 14). The first researcher was EAS, who is a GP–researcher (FRACGP) with a PhD in health services research, and the second researcher was NM, who is a research assistant and medical student in their penultimate year. Analysis was conducted according to the principles of constant iterative comparison.^28^ First, EAS performed a primarily semantic, rather than latent, analysis of the entire dataset without extraneous input. This means that themes were found explicitly within what the participants said and did not extend to underlying ideas or assumptions. Their development of an initial set of codes was guided by the primary research questions and the broader literature. This set of codes was then given to NM. NM read through interview transcripts several times to familiarise themselves with them before completing a first pass of coding, informed by EAS’s codes. After this, EAS and NM met and discussed their codes until agreement was reached about an integrated set of categories and codes that provided a clear conceptual understanding for the aim of this study. These results were then discussed in a meeting with the entire team. Next, the codes were refined by EAS and NM. A series of conversations then took place between EAS and NM, and the broader team of researchers, which achieved consensus about four final categories and their respective sub-codes.

Although we have a small sample of patients, there were strong and consistent messages in the coding and theme development, allowing a rounded understanding of the research objectives.^29^

## Results

Nine patients from a total of 43, who consented to have their consultation recorded, were interviewed. Most patients were female, aged between 25 and 84 years, and living with a chronic disease for >5 years (Table 1). Four GPs (*n* = 2 male, *n* = 2 female) from different clinics agreed to participate in the study. All were aged ≥45 years and had been practising for >20 years.

**Table 1.** Demographic characteristics of nine patients who attended a general practice clinic with a low socioeconomic background (2021–2022)

Patient number	Sex	Age, years	Type of low SES status	Length of living with a chronic disease, years	GP
P1	Female	45–54	PHL	>10	GP1
P2	Male	45–54	P	NA	GP1
P3	Female	35–44	PHL	>10	GP1
P4	Female	75–84	P	5–10	GP2
P5	Female	75–84	P	NA	GP3
P6	Female	25–34	U	>10	GP4
P7	Female	45–54	P	5–10	GP4
P8	Female	65–74	H	5–10	GP3
P9	Female	65–74	P	>10	GP3

H = healthcare card holder. L = live in low-income household. NA = not available. P = received government pension. SES = socioeconomic. U = unemployed.

In addition to the usual difficulties of capturing 'harder-to-reach' populations, the sample size was restricted by strict pandemic restrictions in Melbourne, Australia, across 2021 and 2022. In the video-recordings, GPs and patients wore masks and sat in chairs, distanced apart. In interviews, none of the patients commented on the masks worn or the distancing.

The following four categories were developed relating to: 1) the doctor’s demeanour and the patient’s feelings; 2) the therapeutic relationship; 3) the doctor’s therapeutic skillset; and 4) communication techniques (Table 2). In each of the interviews, the discussion about the video-recorded consultation often triggered memories and reflections about other previous consultations with the respective GP.

**Table 2.** Key categories and respective sub-codes for what makes up a good consultation experience from the perspective of patients from a low socioeconomic background

Categories	Codes
The doctor’s demeanour and how the patient was made to feel during the consultation drove their engagement	Doctor’s demeanour or patient’s feelings Doctor is easy to talk to Patient feels comfortable Patient feels safe Patient feels listened to by doctor Doctor is caring in attitude Doctor is patient Friendly and personable attitude by doctor
An established and collaborative therapeutic relationship was of high importance to patients	Therapeutic relationship Attentiveness by doctor to details and needs of patient Can laugh together Consistency by doctor across consultations Long therapeutic relationship Trust
	Patient-centred management Doctor advocates for patient Checks on patient outside of working hours Follows up on previous management decisions Patient involved in management decisions Creation of realistic management plans
A doctor’s therapeutic skillset was integral to patient confidence and comfort	Therapeutic skillset Communicative with specialists Positive health outcome from management decision Offers referral Doctor engages with difficult topics Positive perception of doctor’s competency Positive perception of doctor’s thoroughness
Patients appreciated verbal and non-verbal communication techniques	Verbal techniques Clear and understandable explanations Thorough explanations Patient feels comfortable asking questions Doctor willing to repeat information as needed
	Non-verbal techniques Use of computer Showcasing they are listening Showcasing they have time Using visual aids in consultation

### The doctor’s demeanour and how the patient was made to feel during the consultation drove the patient’s engagement

Generally, patients reported that when there were behavioural expressions of friendliness, care, and patience from the GP, they felt comfortable to engage with their GP. The importance of friendliness was that it made the patient feel humanised beyond their medical needs:

*‘He makes you feel as if he’s your friend … and you feel as if* [he] *is actually interested in you. Not just interested in fixing your problem and a medical thing. He’s interested in you as a person and I think that’s terribly important.’* (P4)

The interviewer attempted to unpack this friendliness with each patient, but it was rare that the patient could articulate exactly how and why the GP made them feel that they were friendly and a safe person to talk to. Patients perceived their doctor as genuinely caring when they expressed empathy towards medical and non-medical issues. Greater therapeutic trust was fostered when GPs were perceived as caring because patients felt both understood and listened to:

*‘The best thing about him is he listens to you. He really listens. And he really relates to you, and you think he really cares*.*’* (P4)

The demonstration of patience, by not making the patient feel rushed, furthered this sense of trust:

*‘She was patient enough to let me get to the point … like a previous GP* [was] *just rush, rush, rush and I felt like I was wasting their time. Whereas* [Dr.X] *was just patiently waiting. So, it was easier to find the point and get to the point. I didn’t feel rushed.’* (P1)

The product of greater therapeutic trust was that patients felt safer with their GP and therefore more comfortable to communicate their feelings and needs:

*‘She makes me feel comfortable. Like I can tell her* [things] *that I’d normally not want to mention to anyone. You know she has that kind of personality I suppose.’* (P3)

Patients’ comfort in expressing their needs was also driven by the ease with which they felt they could talk to their doctor. The GPs that were perceived as patient, friendly, and good listeners were described as easy to talk to:

*‘It’s as if that you feel you can say anything. He’s just so — he’s the most amazing doctor. He really is. He’s the most amazing doctor. I don’t think I’ve ever had a doctor that’s quite as easy to talk to.’* (P4)

### An established and collaborative therapeutic relationship was of high importance to patients

The presence of a strong therapeutic relationship, which beneficially affected management decisions, was a key aspect of the consultation experience. Many patients correlated their positive attitude towards their doctor with a sense of trust and being known over time. This trust was driven by the doctor’s consistency in demeanour and perceived clinical competency across consultations, which allowed patients to predict how they were going to act, explain topics, and make decisions:

*‘*[I see Dr X instead of others] *mainly because I’ve been seeing* [Dr X] *for years and years and years. So, she knows my medical history, and I feel as if I can talk to her.’* (P8)

Patients had a higher perception of a genuine interpersonal relationship, and the relatability of their GP, when there was attentiveness to their personal details and the ability to be light-hearted and laugh during consultations:

*‘We get* [stuff] *done. And we have a laugh. Yeah, yeah, yeah, the issues I have aren’t hysterical. Like but if we can have a giggle and yeah it’s that person, like having that, like sort of shared interest or common ground or mutual feeling that stops a doctor or someone from being like a position of authority to being someone that I can relate to and trust.’* (P6)

Aside from interpersonal factors, the second element of the therapeutic relationship was the degree of collaboration and patient centredness in management decisions. A sense of collaboration was created by the GP providing the patient with different management options and asking questions about their preferences. Furthermore, the joint creation of realistic management plans helped patients feel more in control over their health:

*‘I trust what she says, you know, she gives me the option … She provides the information, she gives me the options; tells me what’s better and then leaves it up to me.’* (P9)

Patients commented on the extent of follow-up by GPs, either within consultations, future consults, or out of hours. They also valued the GP advocating for them within the healthcare system:

*‘You know you go, maybe, you’ve got a bad chest, well then you tell her that. And* [when] *you go back, she’ll say how are you going with your tablet? How’s your chest? Let’s listen. She’ll do follow-ups … she does bring it up next time.’* (P5)

### A doctor’s therapeutic skillset was integral to patient confidence and comfort

Importantly, all the interviewees spoke highly of their GP’s clinical skillset and described it with two key adjectives: thoroughness and competency. Thoroughness was based on the use of consultation time, depth of engagement with patient concerns, and attention to detail in management plans. The patient’s perception of competence was related to the perceived thoroughness and the GP’s clinical medical knowledge:

*‘I think that it’s a good consultation. I like that my doctor is quite thorough. She looked at different things at different times and she addressed what I am wondering about.’* (P1)

Moreover, patients particularly emphasised two key clinical skills within their general reflections. The first was a readiness to offer referrals. This demonstrated the GP’s knowledge of when it was necessary to involve allied health and specialist care, which made patients more confident in their overall capabilities. The second was the ability to communicate and collaborate with specialists, which made them feel that their GP was coordinating their care and supporting them in navigating the healthcare system:

*‘But he doesn’t hesitate to refer you to someone if he doesn’t know. He immediately refers you to someone. He doesn’t pretend he knows things that he doesn’t.’* (P4)

A positive perception of clinical capabilities was shaped by two other factors, the first being if the GP had made a management decision and this had improved patient’s symptoms. Moreover, if they demonstrated communication-based therapeutic skills, such as sensitively raising and discussing challenging topics, this reinforced the patient’s perception of their overall competency:

*‘It really hits home* [when she raised how I had been feeling low] *because I try very hard not to let anybody see … I can’t say how I feel can I? ... So I was glad when she said it, because she was thinking what I was thinking, so it made me not feel as guilty.’* (P5)

### Patients appreciated verbal and non-verbal communication

Interviewees frequently discussed the quality of their GPs’ explanations. The two most pivotal aspects of an explanation was that it was clear and in-depth. Such explanations enabled patients greater understanding of their health conditions:

*‘She comes through very clear. You know what you need to do. She speaks in a way you can understand what’s going on and what you’re expected to address. So, you know it’s pretty good,* [pretty] *excellent.’* (P2)

When the GP used aids to assist in explanations, whether pictures, diagrams, or 3D models, patients described this as an excellent means to simplify information and increase understanding:

*‘*[Showing the weight chart on the computer] *is an excellent way to show information because I can see how far I am going, my progress with my weight and my health. It’s very exceptional yeah. She explained very well so that I can understand that you know.’* (P2)

The coupling of these verbal and non-verbal techniques promoted collaborative management because patients understood their diagnoses and test results. Enhancing patient understanding was further supported when the doctor was willing to repeat information as needed:

*‘I just appreciate that* [Dr X] *like, explains it again. Because like he’s had to explain it to me about 15 times. So that’s good.’* (P6)

When the GP tailored their communication to the individual and sought to comprehensively explain health-related issues, patients, in turn, felt more comfortable asking questions:

*‘Whereas like I say, if I see* [Dr X] *or* [Dr Y] *they will explain as best they can what’s going on. And that’s why I ask the question … so yeah, I believe in asking questions and hopefully getting the answers I want.’* (P8)

A non-verbal communication technique identified across all interviews was how the GP positioned their upper body relative to their computer screen and how they used the computer. When this was done in a way that showed they were listening, see video descriptionor quote below, the patient felt humanised and of importance:

*‘*[I felt] *comfortable. Better than somebody just starting at a computer, and you’re sat there. She talks to you one-on-one to you, you know like you’re a person and not just in the computer.’* (P5)

Video description: GP is sitting upright in their chair, slightly leaning forward, with their feet and upper body angled towards the patient. Patient is seated at a 90-degree angle to computer, which is directly in front of the GP. GP isn’t typing on the computer, but rather positioned as above and listening to the patient. The GP is nodding along as the patient speaks.

Patients noticed and appreciated other non-verbal body language cues, including head nods and shoulder movements as outlined in the video descriptions below, which communicated that the GP was engaged with the consultation and had time to discuss their health needs:

*‘Yeah, notice the nod then, watching that, it’s like she’s really listening and taking on board and acknowledging and agreeing.’* (P3)

Video description: Doctor is looking directly at patient, her elbow resting on the table, hands laid on top of each other and shoulders pointing towards the patient. The patient is talking, answering the GP’s question. Throughout her entire answer the GP maintains eye contact, doesn’t type notes, or look at the computer. As the patient says key points the GP responds with a nod, while maintaining eye contact.

*‘How calm she is kind of makes me calm as well. Yeah, she’s not rushed. She’s calm and just going through the motions and so it makes me calm … Like her voice is calm and her body is calm I think.’* (P3)

Video description: Patient raised a new symptom that had been troubling them towards the end of the consultation. The GP asked several questions about it and expressed no indications of being in a hurry. They then slowly got up and examined the patient, reassured them, and discussed what might be causing it alongside management options. They asked for their input on the management plan, and then arranged follow-up.

## Discussion

### Summary

The aim of this explorative pilot study was to investigate what creates a positive GP consultation from the perspective of patients from a low SES background. The unique time period of intense pandemic restrictions, alongside the usual challenges of recruiting ‘harder-to-reach’ individuals, led to a small sample of patients. Yet by interviewing individuals from this patient group, while they watched video-recordings of their consultation, the importance of what can be described as an established and collaborative therapeutic relationship was highlighted. Interviewees also reflected on their previous experiences with the GP and did not isolate their discussion to the single-recorded consultation, but rather emphasised factors between a doctor and patient that are demonstrated and reinforced over time. Specifically, patients reported that a GP’s interpersonal demeanour and communication skills were critical for a positive experience. Overall, this sample of patients’ experiences were shaped by: 1) the doctor’s demeanour and the patient’s feelings; 2) the therapeutic relationship; 3) the doctor’s therapeutic skillset; and 4) communication techniques.

### Strengths and limitations

A strength of this research was that the video-stimulated-recall interviews limited recall bias. This increased the credibility of our results and allowed for greater understanding of the patient perspective. Also, our appreciative enquiry approach meant that we interviewed patients whose consultation experience was good, based on the parameters of feeling listened to, respected, and having adequate time with their GP. This is both a strength, in that it supports our method, and limitation, in that it may have resulted in selection bias of this research. Regarding limitations, our study is somewhat restricted in transferability because of the small sample size (four GPs and nine patients) and limited diversity in age and sex (the latter in this study was one male patient and eight female patients), which resulted from recruitment challenges relating to COVID-19.

### Comparison with existing literature

An established therapeutic relationship, through continuity of care with a practitioner, appears to be a key driver of positive consultation experiences for our sample of participants. This finding is mirrored in research that positively correlates the presence of relational continuity and the degree of patient satisfaction both generally,^19,30–32^ and within primary care.^15,33,34^ Relational continuity of care provides a sense of security,^35^ because the established relationship enhances trust, engagement, and understanding.^15^ In their work on patients living in deprived areas of Scotland, Mercer *et al* established that, according to the patient, continuity in primary care added to consultation quality.^15^ Although this pilot study was small, our findings are similar to that of Mercer *et al.* This study extends Mercer *et al*'s findings by identifying the need for continuity in care specifically in patients who are from a low SES background with chronic health needs. Previous qualitative research has also highlighted the importance of relational continuity of care for patients with chronic health problems.^36^

Current literature affirms our emergent category of the doctor’s therapeutic skillset and its potential to be positively associated with patient satisfaction regarding consultation quality.^15,19,37^ Similarly to Fung and Mercer^37^ and Mercer *et al*,^15^ the technical competency of the doctor in the present study seemed to be intertwined with the overall consultation quality, because of how the former generates confidence in the doctor, with this bi-directional relationship also influenced by past experiences. Interviewees appeared to value thoroughness by their GP, especially when engaging with problems raised, and attributed it to a good technical skillset. This has been reflected in non-deprivation primary care consultation literature, where completion of physical examinations by doctors indicated thoroughness and commitment to gaining a holistic understanding of the problem.^37^ While this study was exploratory, our findings provide insights into what is perceived as a good clinical skillset, beyond positive diagnostic outcomes, for low SES groups when discussing consultation quality.

In this study the GP’s interpersonal demeanour encompassed affective behaviours that demonstrated care, including empathy and friendliness. These findings are reflected in the literature,^38,39^ with a systematic review identifying the degree of physician interpersonal care as the main determinant of patient satisfaction.^19^ Specifically within the primary care literature, patients from low SES backgrounds identified empathetic concern as contributing to consultation quality,^15^ with the degree of physician empathy, in areas of deprivation, being associated with patient enablement post-consultation.^40,41^ In our sample, the patients raised that friendliness within the consultation helped to humanise the interaction and increase GP relatability. This may add to the current distinct role of friendliness, separate to empathy, in shaping consultation experience. While communication skills have been associated with patient satisfaction within the consultation experience both generally,^19^ and for low SES groups,^15^ to the best of our knowledge, the emphasis interviewees placed on non-verbal communications has not been identified within low SES primary care previously. However, the presence of positive non-verbal communication has been correlated to a higher sense of clinician empathy;^42^ hence, in this study, non-verbal communication may have supported a positive perception of the GP’s expression of empathy, both shaping the overall consultation experience. Our methodology may have supported identifying these findings because patients were able to reflect on their recorded consultation.

### Implications for research and practice

The exploratory nature of this pilot study limits our ability to propose adaptations to current practice to improve the GP consultation experience for patients from low SES backgrounds. However, there are several findings that the authors feel are important to highlight. First, given the importance of patient satisfaction in engagement and health outcomes, this study reinforces existing research, which highlights the need for primary care policies that address inequities in accessing and promoting therapeutic relational continuity in high deprivation areas, which includes supporting GP retention.

Our results highlight the positive impact that interpersonal care and communication skills can have on the consultation experience of patients from low SES backgrounds. For new interventions in primary care, these high-level verbal and non-verbal communication factors could be considered, alongside other strategies, to assist in reducing the differences in consultation experiences for those in low SES groups. This may, in turn, influence health outcomes for this group because high-quality therapeutic relationships and consultations are believed to be imperative to meeting needs of vulnerable people groups, and equipping them to effectively use primary health care.^5,20^

Additional work is needed, with a larger, more diverse participant group, to understand the GP consultation experience of low SES groups, and how this differs based on other determinants of health including ethnic group and geographic location. Within this it may be appropriate to consider including a comparison group, as in those from non-disadvantaged backgrounds, to nuance further clinical implications for patients from low SES groups and other disadvantaged backgrounds more broadly.

In conclusion, this exploratory study identified four major categories that positively influence GP consultation experiences for patients from low SES backgrounds. Our qualitative methodological model offers detailed insights into the individual perspectives of this ‘hard-to-reach’ group on consultation quality. The emphasis interviewees placed on relational continuity of care, empathetic care, and communication skills could inform future research that, in turn, may be able to provide more nuanced and transferable clinical practice recommendations. This research is a small but important step towards increasing our understanding of the experience of individuals from low SES backgrounds in primary care and the existing health inequities within this area.

## Data availability

The data underlying this article will be shared on reasonable request to the corresponding author.
